# A commentary on ^125^I seed brachytherapy for refractory loco-regional recurrence of non-anaplastic thyroid cancer

**DOI:** 10.3389/fonc.2022.932001

**Published:** 2022-08-11

**Authors:** Yamei Wang, Haiyang Wang, Huawen Xia

**Affiliations:** Department of Interventional Surgery, Handan First Hospital, Handan, China

**Keywords:** non-anaplastic thyroid cancer, recurrence, brachytherapy, efficacy evaluation, safety evaluation

In a recent retrospective single-center study, Yu et al. (1) treated patients with recurrent non-anaplastic thyroid cancer using iodine-125 (^125^I) seed brachytherapy and achieved great therapeutic effects. The results of this study carry significant implications for the treatment of refractory loco-regional recurrence of non-anaplastic thyroid cancer. However, the study has some problems that we would like to discuss with the authors.

First of all, the authors included only 15 patients over nearly 13 years. Such a sample size is too small to be representative of the entire disease population. During the follow-up period, a total of 10 (66.7%) patients did not reach the follow-up endpoint, which can seriously affect the credibility of the results. We recommended the authors increase the sample size and extend the follow-up period to draw more reliable conclusions.

Secondly, there are some statistical errors in the article. The calculation of median follow-up time is not simply the median follow-up time of each patient, but the value obtained by inverting the value of endpoint events and censored events when calculating median survival time by the Kaplan–Meier method. Clearly, the authors directly took the median follow-up time of each patient (48 months) as the median follow-up time. We recalculated the median follow-up time based on the data provided by the authors in Table 2, and the result was 64 months instead of the 48 months as described by the authors. In addition, the overall survival (OS) curve in the paper is inconsistent with the data in Table 2. As we can see, there are altogether 10 censored data in the OS curve, while there are only seven cases of DWD patients in Table 2, that is, the remaining eight cases are censored data. The censored data are inconsistent. I wish the authors could re-examine the raw data carefully.

Thirdly, the baseline of patients included in the article varied widely, which might influence the results. For example, patients with poor cardiopulmonary function eventually died of heart failure rather than thyroid cancer, which would lead to an underestimate of the efficacy of ^125^I on the prognosis of the patient. Therefore, we suggest that the authors should focus on patients’ disease-specific mortality rather than OS (2).

Lastly, as described in the article, patient 9 experienced five sessions of brachytherapy in a year, and the activity of the ^125^I seeds used in this patient ranged from 0.3 to 3.0 mCi. Why such a wide range of activity of the seeds are used in the patient? What is the basis for the selection of seed activity? The occurrence of radioactive damage may be increased by 3.0 mCi seeds (3). In addition, how to calculate the radiation absorbed dose of tumors treated by repeated multiple seed implantations is still a challenge. The absorbed dose of the tumor should not be a simple addition of EQD2 because the continuous shrinkage of a tumor will lead to concentric aggregation of seeds, and the actual absorbed dose will theoretically be larger than the calculated EQD2 (4,5).

In conclusion, the sample size of the article is small and there are many censored survival data in the article, which can seriously affect the credibility of the results. So the author is suggested to further expand the sample size and extend the follow-up time to draw more reliable conclusions. Moreover, we suggest the authors should focus on patients’ disease-specific mortality rather than OS. Finally, there are some statistical errors in the paper, so the author is expected to check it again.

## Author contributions

YW, HW, and HX wrote the letter. All authors contributed to the article and approved the submitted version.

## Acknowledgments

The authors would like to thank the participating members. This research did not receive any specific grant from funding agencies in the public, commercial, or not-for-profit sectors.

## Conflict of interest

The authors declare that the research was conducted in the absence of any commercial or financial relationships that could be construed as a potential conflict of interest.

## Publisher’s note

All claims expressed in this article are solely those of the authors and do not necessarily represent those of their affiliated organizations, or those of the publisher, the editors and the reviewers. Any product that may be evaluated in this article, or claim that may be made by its manufacturer, is not guaranteed or endorsed by the publisher.
